# p53 Down Regulates PDGF-Induced Formation of Circular Dorsal Ruffles in Rat Aortic Smooth Muscle Cells

**DOI:** 10.1371/journal.pone.0108257

**Published:** 2014-09-23

**Authors:** Laura J. Payne, Robert L. Eves, Lilly Jia, Alan S. Mak

**Affiliations:** Department of Biomedical and Molecular Sciences, Queen's University, Kingston, Ontario, Canada; Universität Regensburg, Germany

## Abstract

The tumor suppressor, p53, negatively regulates cell migration and invasion in addition to its role in apoptosis, cell cycle regulation and senescence. Here, we study the roles of p53 in PDGF-induced circular dorsal ruffle (CDR) formation in rat aortic smooth muscle (RASM) cells. In primary and immortalized RASM cells, up-regulation of p53 expression or increase in activity with doxorubicin inhibits CDR formation. In contrast, shRNA-knockdown of p53 or inhibition of its activity with pifithrin α promotes CDR formation. p53 acts by up-regulating PTEN expression, which antagonizes Rac and Cdc42 activation. Both lipid and protein phosphatase activities of PTEN are required for maximal suppression of CDR, but the lipid activity clearly plays the dominant role. N-WASP, the downstream effector of Cdc42, is the major positive contributor to CDR formation in RASM, and is an indirect target of p53. The Rac effector, WAVE2, appears to also play a minor role, while WAVE1 has no significant effect in CDR formation. In sum, we propose that p53 suppresses PDGF-induced CDR formation in RASM cells by upregulating PTEN leading mainly to the inhibition of the Cdc42-N-WASP pathway.

## Introduction

Cell migration and invasion of the extracellular matrix (ECM) play critical roles in cross-tissue migration of vascular smooth muscle cells in atherosclerotic plaque formation and stability, and cancer cells during metastasis [1], [2]. Although cell invasion and cell migration are intimately related processes, they require different cytoskeletal organelles regulated by distinct mechanisms. For instance, digestion of the ECM by invasive cells requires the formation of specialized invasive, actin-based organelles such as podosomes and invadopodia [3]. Cell migration, on the other hand, requires a different set of cytoskeletal organelles: focal adhesions, filopodia, lamellipodia, and circular dorsal ruffles (CDR).

Unlike other dynamic membrane structures such as lamellipodia and filopodia that assemble and disassemble repeatedly after stimulation, CDRs appear only transiently upon growth factor stimulation with a life span of 5-30 min [4]–[6]. They emerge as ring-shaped waves spreading quickly across the dorsal cell surface, followed by ring closure and collapse inside the cell. A single cell often harbors more than one ring; but for unknown reasons, CDRs do not form again after 30–45 min of stimulation even though the stimulant remains active in the media. One of the proposed functions of CDRs is to initiate movement in immobile cells, but they may also play significant roles in macropinocytosis, ECM degradation, cell polarization, and internalization of receptor tyrosine kinases [4], [7]. Recently, CDR have been implicated in the internalization of integrins through macropinocytosis, and translocation of integrins from disassembled focal adhesions at the rear to newly formed focal adhesions at the leading edge of the cell [8].

The mechanism by which CDR formation and disassembly are regulated is not fully understood. Platelet-derived growth factor (PDGF), a potent chemoattractant for VSMC [9], [10], is an effective agonist of CDR formation commonly employed in *in vitro* studies. Acting downstream of the PDGF receptor, the RacGTPase is one of the major regulators of CDR formation [7], [11]–[13]. Rac has been shown to be required to produce CDR, by activating the WASP family members, WAVE1/2, and the Arp2/3 pathway leading to branched actin filament formation in membrane ruffles. Furthermore, loss of WAVE1, not WAVE2, impairs CDR formation [14]. However, it has also been suggested that N-WASP, but neither WAVE1 nor WAVE2 (also called Scar1 and Scar2, respectively) is important in robust CDR generation in mouse embryonic fibroblasts [15], implicating the involvement of Cdc42.

The tumor suppressor, p53, better known for its roles in the regulation of cell cycle progression and apoptosis, has been shown to play a significant role in suppressing cell motility and invasion [16]–[20]; however, the detailed mechanisms involved have not been deciphered. Although Rac is not a transcription target of p53, it has been shown that p53 down regulates Rac activity in cell migration [21], [22] and inhibits lamellipodia formation [13]. p53 also suppresses cell invasion by inhibiting podosome formation in Src-transformed RASM cells and fibroblasts [23].

In this study, we have investigated whether p53 plays a role in the regulation of PDGF-induced CDR formation in RASM cells. We have found that p53 suppresses CDR formation in primary and immortalized RASM cells. Furthermore, we have shown that p53 acts by up-regulating PTEN, which in turn suppresses the Cdc42-N-WASP pathway.

## Materials and Methods

### Plasmid constructs, shRNA, and siRNA

Wild-type murine p53 (wtp53) was generated as previously described [24]. The expression construct for wild-type PTEN (wtPTEN) (MMM1013-7511653) was purchased from Open Biosystems. All short hairpin RNAs (shRNAs) were generated using a mir-30-based design method, which has been previously described [25]. A TMP or LMP vector system (Open Biosystems) was used for the cloning and expression of the shRNAs [26]. Each shRNA sequence could target both rat and mouse transcripts. Two shRNAs were generated for p53 (rat NM_030989 and mouse NM_011640). The target/sense sequences used to design the shRNAs were 5′-GTC(A/T)GGGACAGCCAAGTCTGT-3′ and 5′-CG(T/C)GCCATGGCCATCTACAAG-3′ [26]. Two shRNAs were generated for PTEN and the target/sense sequences used to design the shRNAs were 5′-GAGATCGTTAGCAGAAAGAAAA-3′ and 5′-CCACAGCTAGAACTTATCAA-3′ [23]. Mutations for the C124SPTEN mutant (phosphatase dead), G129EPTEN mutant (protein phosphatase only) and Y138LPTEN mutant (lipid phosphatase only) were made using the QuikChange II XL site-directed mutagenesis kit (Stratagene), as previously described [23].

Smartpool siRNA mixes for Rac1, WAVE1, WAVE2, N-WASP and Cdc42 to Rat were purchased from Dharmacon. Negative control 1 FAM-labeled siRNA was used, which does not correspond to any sequence in the human, rat or mouse genomes (Ambion) [27].

### Cell culture and creation of stable cell lines

Protocol for isolation of rat aorta was approved by Queen's University Animal care committee protocol #Mak-2006-010-OR1. Rats were anaesthetized with sodium pentabarbitol before dissection to ameliorate suffering [26], [28]. RASM were cultured in high glucose Dulbecco's modified Eagle's medium (DMEM) (Invitrogen), supplemented with 1% penicillin G/streptomycin sulphate (1%P/S) (Invitrogen) and 10% bovine growth serum (BGS) (HyClone). Cells were grown in an incubator at 37°C in the presence of 5% CO_2_. Cells were maintained at sub confluent levels (approximately 80–90%). Creation of retroviral cell lines was carried out as previously described [26]. Post-infection selection on cell lines was performed using 5 µg/mL of Puromycin (Fisher Scientific) or 1 mg/mL Neomycin (Sigma-Aldrich), based on cell type.

### Transient transfections

Glass coverslips (Fisher Scientific) were coated with 5 µg/mL human fibronectin (Roche Applied Science) and placed in 24 well plates for two hours. SMC in DMEM 10%BGS 1%P/S were plated at a density of 10 000 cells/well. After 24 hours of incubation, the cells were transfected with 0.8 µg/well of the appropriate DNA in serum free media, following the Lipofectamine 2000 protocol (Invitrogen). After four hours, the media was changed to DMEM 10%BGS 1%P/S. siRNA transfection were done using Dharmafect 2 (Dharmacon) as per manufacturers protocol.

### Pifithrin α and Doxorubicin treatment

Glass coverslips (Fisher Scientific) were coated with 5 µg/mL human fibronectin (Roche Applied Science) and placed in 24 well plates for two hours. RASM cells in DMEM 10%BGS 1%P/S were plated at a density of 10 000 cells/well. After 24 hours of incubation, the media on the cells was switched to serum free media and the cells were incubated for an additional 24 hours. The p53 inhibitor, pifithrin α (PFA) (20 µM) (Sigma-Aldrich) or the genotoxic drug doxorubicin (Adriamycin) (500 ng/mL) (Sigma-Aldrich) was added to the wells and incubated for 24 hours [26].

### PDGF treatment

Approximately 16 hours after seeding cells, media was replaced with serum-free DMEM. After approximately 24 hours of incubation, 20 ng/µL PDGF-BB (Sigma-Aldrich) diluted in serum-free media was added to the coverslips. After 20 min of incubation, the cells were immediately fixed, permeabilized, and stained as previously described [29].

### Antibodies and dyes

The following primary antibodies were used in this study for immunofluorescence microscopy and western blotting: c-myc (Sigma-Aldrich), cortactin 4F11 (Millipore), Glyceraldehyde-3-Phosphate (GAPDH) (MAB374) (Millipore), p53 (Cell signaling), Rac1 (Cell Biolabs), PTEN (Cell Signaling), Cdc42 (Cell Biolabs), WAVE1 (Abcam), WAVE2 (Abcam), N-WASP (Cell signaling), and MDM2 (M4308) (Sigma-Aldrich), GTP-Rac (Neweast Biosciences). Alexa Fluor 488- and Alex Fluor 568-conjugated secondary antibodies (Molecular Probes). Tetramethyl rhodamines isothiocynate (TRITC)-conjugated phalloidin (P1951) (Sigma-Aldrich) was added along with the secondary antibodies to stain for F-Actin. Western blotting was performed using the primary antibodies cited above as well as specific anti-mouse and anti-rabbit horseradish-peroxidase conjugated secondary antibodies (Millipore).

### CDR imaging and quantification

Immunostained cell imaging and live cell imaging were performed using a Zeiss AxiovertS100 microscope equipped with a Plan-Neofluar 40X/0.75 objective, high-performance charge-coupled device camera (SensiCam; Cooke Corp.). Images were analyzed using Slidebook image analysis software (Intelligent Imaging Innovations), ImageJ software (NIH), Corel PhotoPaint software, and Image Pro Plus software (MediaCybernetics). A cell is defined as having dorsal ruffle if one or more rings containing both actin and cortactin appear on the dorsal surface of the cell. RASM cells were cultured on fibronectin coated ΔT dishes and serum starved overnight. Media was replaced with DMEM supplemented with HEPES buffer (Invitrogen) and 20 ng/mL PDGF. Time-lapse images were taken every 30 seconds.

### Statistical Analysis

Statistical analysis was performed using data from 3 separate experiments where 150–200 cells per experiment were counted. Bars represent standard deviations calculated from the 3 experiments. The p value was calculated using a 2-tailed student T-test. Data sets were deemed statistically significant if the p value was <0.05 and indicated by * or # as described in figure legends.

## Results

### p53 inhibits the formation of circular dorsal ruffles

**Figure 1:**
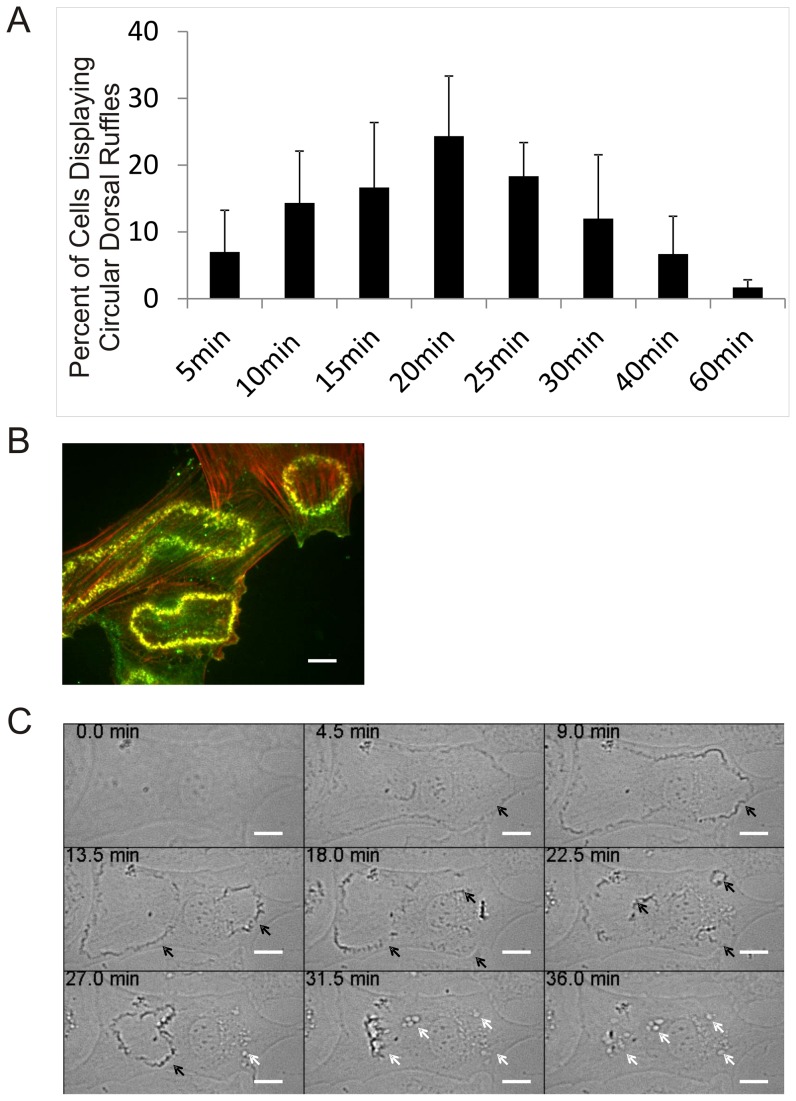
PDGF induces circular dorsal ruffles in RASM cells. (A) Primary RASM cells at passage 4-6 were cultured on fibronectin coated coverslips and serum-starved overnight then treated with 20 ng/ml PDGF for different times. At least 200 cells were counted for each time point. The average percentage of cells that produced CDR was obtained from 3 experiments. Error bars represent standard deviation from 3 experiments. (B) Representative fluorescence microscopic image of CDRs formed in cells treated with 20 ng/ml PDGF for 20 min. CDRs are identified as yellow rings where cortactin (green) is identified by immune-fluorescent stain and F-actin (red) by fluorescent phalloidin. Scale bar represent 20 µm. (C) Time-lapse phase microscopic images of primary RASM cells treated with 20 ng/ml PDGF. Black arrows indicate CDRs, white arrows show macropinosomes formed upon CDR closing. Scale bar represent 20 µm. See Movie S1 for video.

We first established that primary RASM cells produce CDR in response to PDGF stimulation as has been observed in MEF cells. As shown in Fig. 1A, primary RASM cells were treated with 20 ng/mL of PDGF for different times and the percentage of cells that produced at least one CDR were counted. At the optimal time point of 20 min, 15–30% of cells produced CDR, with an average life span of 10 min. In comparison, CDR formation in MEF peaks at 5–10 min of 20 ng/ml of PDGF treatment with 80–90% of the cells forming at least one CDR rings (data not shown). Fig. 1B shows fluorescence microcopy of cells harboring CDRs that contain both cortactin and F-actin.

Phase microscopy (Fig. 1C) shows that PDGF induces a round of CDR formation about 5–20 min after treatment. Each cell may house one or more CDR simultaneously. These CDRs exist as rings for about 5-30 min and coalesce into dense patches that eventually collapse inside the cell with concomitant appearance of macropinosomes. In some of the CDR-producing cells (<10%), this is followed by the appearance and disappearance of another round of CDR (not shown). Even though PDGF still remains in the medium, no more CDR can be detected after 60 min of PDGF treatment. These data suggest that the cells have developed a desensitizing mechanism against prolonged PDGF stimulation.

**Figure 2:**
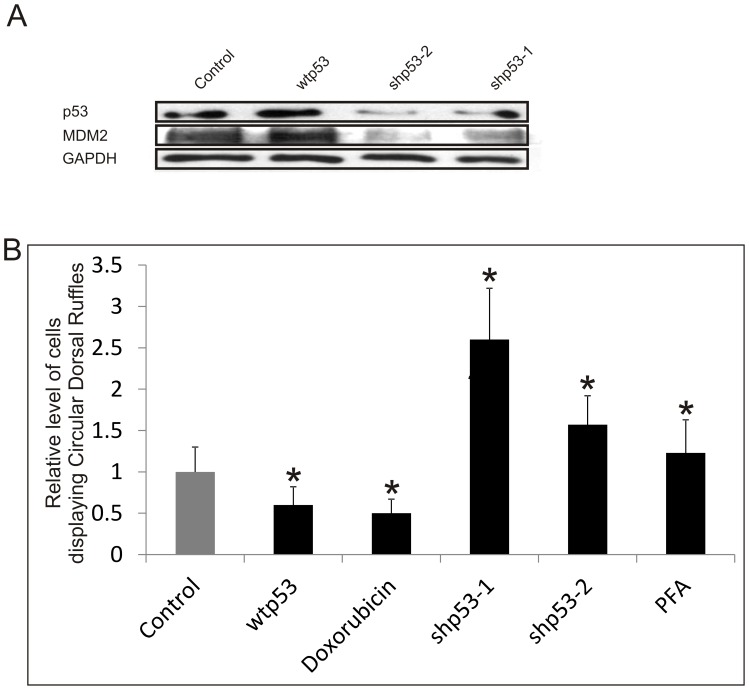
p53 inhibits the PDGF-induced formation of circular dorsal ruffles in immortalized RASM cells. (A) Expression of p53 and MDM2 in immortalized RASM cells expressing the empty retroviral vector, wtp53, shp53-1 or shp53-2 were analysed by Western blots using GAPDH as a loading control. (B) The effects of p53 expression or activity on CDR formation. RASM cells were serum starved overnight and treated with 20 ng/ml PDGF for 20 min. p53 activity was either up-regulated by 500 ng/ml doxorubicin or inhibited by 20 µM PFA for 30 min before and during PDGF treatment. CDRs were fluorescence stained for cortactin and F-Actin (phalloidin). The percentage of cells producing at least one CDR were counted, and normalized to that of the control cells expressing empty vectors or control shRNA. Error bars represent standard deviation from at least 3 separate experiments, * represents p<0.05 with respect to the control cells.

To determine if p53 antagonizes PDGF-induced CDR formation, we expressed wild-type p53 (wtp53) or p53-targeting shRNAs (shp53) in immortalized RASM cells, and used these cell lines to determine possible mechanisms. Stable RASM cell lines were created by retroviral infection that expressed wtp53, two different shp53 (shp53-1 and shp53-2) or a shRNA control that has no homology to any known human mouse or rat sequence. As shown in Fig 2A, and in agreement with our previous findings [26], cells overexpressing wtp53 show an increase in MDM2, the E3 ligase that is a direct transcriptional target of p53 [30], while knockdown of p53 by shRNAs leads to a decrease in MDM2 expression. 15–30% of the PDGF-treated control cells expressing empty retroviral vectors produce CDR, a level similar to that observed in primary RASM cells (Fig. 2B). After 20 min of PDGF treatment, cells overexpressing wtp53 are 40% less likely to produce CDR than control cells (Fig. 2B). Similarly, cells treated with doxorubicin (Doxo), which activates p53 by causing breaks in double-stranded DNA, were 50% less prone to form CDR (Fig 2B). In contrast, knockdown of p53 using two different shRNAs, shp53-1 and shp53-2, resulted in 150 and 60% increase, respectively, in the percentage of cells displaying CDR (Fig. 2B). Inhibition of p53 with Pifithrin-α (PFA) also promotes PDGF-induced CDR formation (Fig. 2B). Together these data show that p53 suppresses PDGF-induced CDR in immortalized RASM cells.

### p53 suppresses Rac- and Cdc42-mediated CDR formation

**Figure 3:**
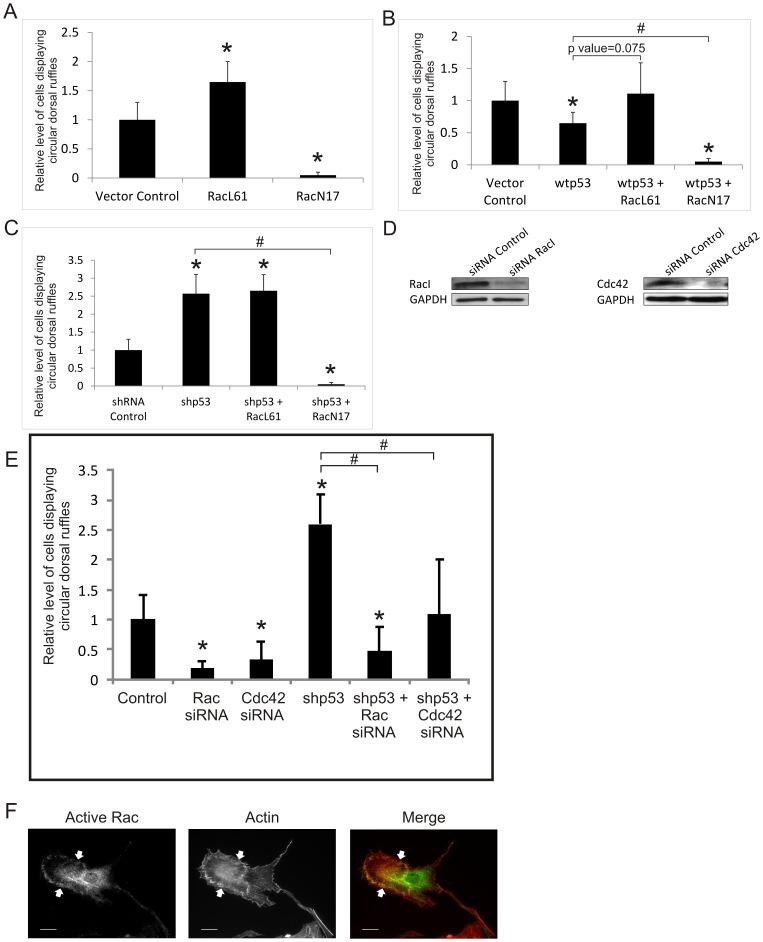
p53 antagonizes Rac and Cdc42 in the regulation of circular dorsal ruffle formation. (A–C) Effects of the constitutively active (RacL61) and dominant negative (RacN17) Rac mutants on CDR formation. Immortalized RASM cells were transiently transfected with RacN17 or RacL61 constructs (A). Immortalized cells overexpressing wtp53 (B) or shRNAs (C) were transiently transfected with either RacL61 or RacN17. (D–E) Effects of siRNA-knockdown of Rac1 and Cdc42 on CDR formation. Western blot analysis of whole cell lysate displays the efficiency of siRNA knockdown of Rac1 and Cdc42 compared to negative control siRNA. GAPDH was used as a loading control (D). RASM cells expressing control shRNA or p53 shRNA (shp53-1) in the background were transiently transfected with Rac1 or Cdc42 siRNAs for 48 hours (E). Cells in (A–E) were treated with 20 ng/mL PDGF for 20 min. The percentage of cells displaying at least one CDR was determined and normalized to that of the respective controls. * represents a p value <0.05, with respect to empty vector control cells, # represents a p value <0.05, with respect to shp53 cells. (F) Active-Rac staining is enhanced in CDRs. RASM cells treated with 20 ng/ml PDGF for 20 minutes were stained for F-Actin (red) and GTP-Rac (green). Colocalization of Actin and GTP-Rac can be seen in CDRs as indicated by white arrows. Scale bar represents 20 µm.

Next, we ask if p53 inhibition of CDR is mediated by down-regulation of Rac. We transiently transfected the immortalized wtp53 and shp53-1 RASM cells with the myc-tagged constitutively active (RacL61), and dominant negative (RacN17) mutants of Rac1. The transfected cells were serum starved for 24 hours and treated with PDGF for 20 min. As shown in Fig. 3A, transient RacL61 transfection causes 60% increase in the propensity of cells to form CDR. On the contrary, RacN17 practically abolishes the ability of the cells to produce CDR. RacL61 is able to restore PDGF-induced formation of CDR in cells that overexpress wtp53 (Fig. 3B), and has no additional effect on the already high potentials in CDR formation of cells expressing shp53 (Fig. 3C). The dominant negative mutant, RacN17, remains a potent inhibitor of CDR in both wtp53 and shp53 cells (Fig 3B and 3C). As shown in Fig. 3F, active GTP-bound Rac1 is localized to CDR. These results suggest that Rac is a possible negative target of p53 in the suppression of PDGF-induced CDR formation.

Since the dominant negative RacN17 mutant acts by sequestering GEFs, some of which may not be Rac specific [31], there is a need to determine whether Rac is the specific target of p53. We therefore examined the effect of siRNA-knockdown of Rac1 on PDGF-induced CDR formation in the vector control and shp53-cells. Transient knockdown of Rac1 (Fig. 3D), reduces the ability of cells to form CDR by 80% (Fig. 3E). In cells expressing shp53-1 in the background, transient knockdown of Rac1 more than nullified the enhancement of CDR production induced by shp53. Although these data show clearly that Rac1 is a key regulator of CDR formation that acts downstream of p53, siRNA-knockdown of Rac is less effective as RacN17, which completely abolishes CDR formation (Fig.3A). These differences may be a result of incomplete Rac1 knockdown or alternatively, RacN17 may have sequestered GEFs of other members of RhoGTPases such as Cdc42. To determine if Cdc42 is also a mediator of CDR formation and a target of p53, we have shown that transient siRNA-knockdown of Cdc42 inhibits CDR formation by about 70% (Fig. 3D and 3E). Furthermore, knockdown of Cdc42 abolishes the effect of shp53 in the promotion of CDR formation (Fig 3E). These data together indicate that p53 suppresses both Rac1- and Cdc42-mediated CDR formation in RASM cells.

### N-WASP is an indirect target of p53

**Figure 4:**
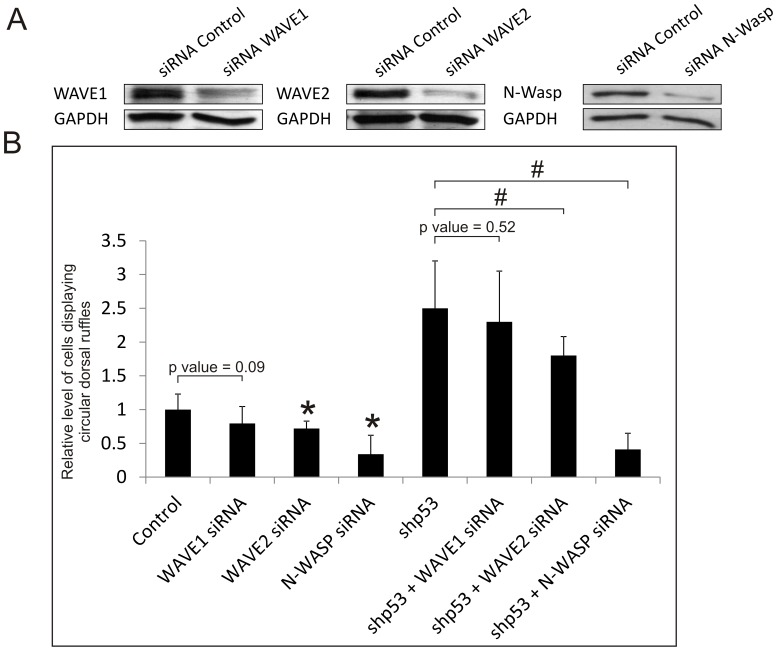
Effects of siRNA knockdown of WAVE1/2 and N-WASP in PDGF-induced circular dorsal ruffle formation. (A) Western blots show the efficiency of siRNA knockdown of WAVE1, WAVE2 and N-WASP. GAPDH was used as a loading control. Viral immortalized RASM cells were transiently transfected with siRNAs to WAVE1, WAVE2, N-WASP or negative control siRNA. (B) RASM cell lines expressing shRNA control (Control) or shp53-1 RNA were transiently transfected with siRNAs targeted to WAVE1, WAVE2 or N-WASP. PDGF-induced formation of CDRs was detected by immunofluorescence staining for cortactin and phalloidin-staining for F-actin. % of cells containing at least one CDR were counted and normalized to the control. Error bars represent standard deviation of at least 3 experiments. * represents a p value <0.05, with respect to control cells, # represents a p value <0.05, with respect to shp53 cells.

Next, we determined whether the downstream effectors of Rac and Cdc42, WAVE1/2 and N-WASP, respectively, are indirect targets of p53. Transient siRNA-knockdown of WAVE1 (Fig. 4A) appears to have a negative effect (20%) on CDR formation, but the data are statistically insignificant (p = 0.09) (Fig. 4B). WAVE2 knockdown has a slightly larger, negative effect on CDR formation (30%) and the data are deem significant (p<0.05). On the other hand, knockdown of N-WASP substantially inhibits CDR formation by over 70% (p<0.05). These results suggest that N-WASP is the major contributor to CDR formation, while WAVE1 and WAVE2 may only play minor roles.

To assess whether N-WASP and WAVE1/2 are agonistic to p53 in CDR formation, we determined if siRNA knockdown of these Arp2/3-activators may blunt the pro-CDR potentials of cells stably expressing shp53. As shown in Fig. 4B, siRNA-knockdown of N-WASP not only nullifies the enhanced CDR formation potentials of the shp53-1 cells, it further reduces CDR formation below that of control cells. To a much lesser extent, WAVE2 knockdown also appears to antagonize the shp53-1 effect on CDR formation, while WAVE1 has no significant effect. Together these data are consistent with the notion that p53 inhibition of PDGF-induced CDR formation in RASM cells is mediated mainly via the Cdc42-N-WASP pathway, and less significantly by WAVE1/2.

### p53 inhibits CDR formation by up-regulating PTEN

**Figure 5:**
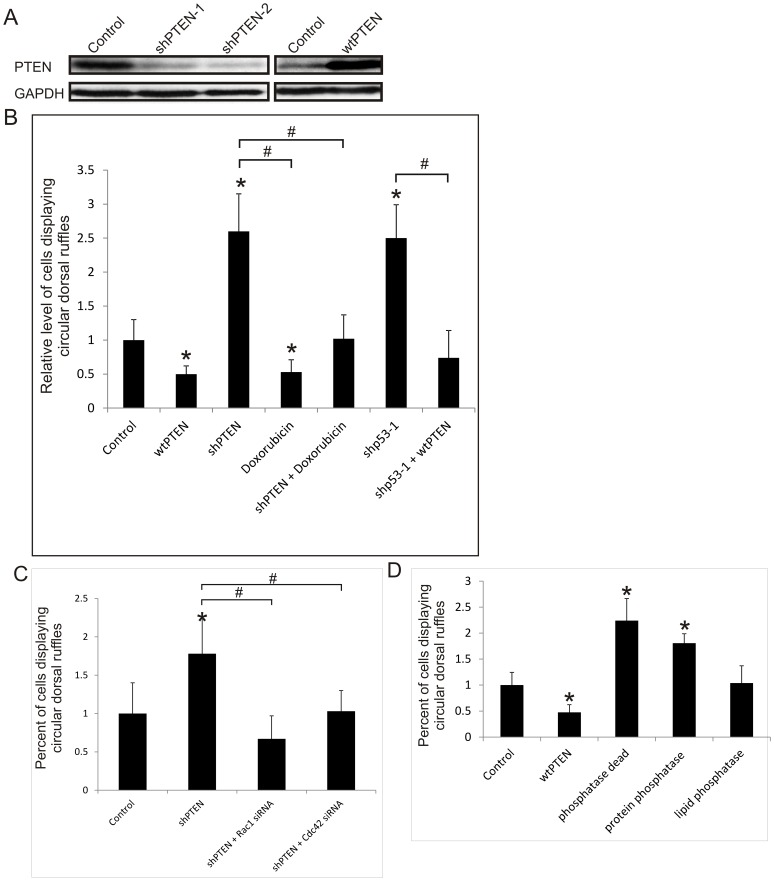
PTEN acts downstream of p53 and inhibits the formation of circular dorsal ruffles. (A) Expression of PTEN in RASM cell lines expressing wtPTEN or shRNAs that target PTEN (shPTEN) was analysed by Western blot. GAPDH was used as a loading control. (B) RASM cell lines expressing wtPTEN, shPTEN, shp53-1 or shRNA control were treated with 500 ng/ml Doxorubicin as indicated; shp53-1 cells were transiently transfected with wtPTEN. (C) RASM cell lines expressing shPTEN or shRNA control were transiently transfected with siRNA to Rac1, Cdc42 or control siRNA for 48 hours. (D) RASM cell lines expressing vector control, wtPTEN, PTENC124S (phosphatase dead), PTENY138L (protein phosphatase dead) or PTENG129E (lipid phosphatase dead). Expression of wtPTEN nullifies the effect of shp53 on PDGF induced CDR formation. RASM cells in (B–D) were treated with 20 ng/ml PDGF for 20 min, and CDRs were fluorescence stained for cortactin and F-Actin (phalloidin). % of cells containing at least one CDR were counted and normalized to the respective controls. Error bars represent standard deviation of at least 3 experiments. * represents a p value <0.05, with respect to control cells, # represents a p value <0.05, with respect to shp53 cells or shPTEN cells as indicated.

Next, we study the mechanism by which p53 down regulates Rac and Cdc42 in CDR formation. Neither Rac nor Cdc42 has been shown to be a transcriptional target of p53; however, p53 may down regulate Rac and Cdc42 by promoting the hydrolysis PI(3,4,5)P_3_, the GEF-activator, via upregulation of PTEN, which has been shown to be a p53 transcriptional target [23], [32]. To determine if PTEN mediates the p53-suppression of CDR formation, stable cell lines were created for RASM cells in which wild-type PTEN had been overexpressed (wtPTEN), or knocked down by shRNAs (shPTEN-1 and shPTEN-2) (Fig. 5A). Fig. 5B shows that overexpression of wtPTEN results in a 50% decrease in the percentage of cells displaying CDR in response to PDGF-treatment, whereas shRNA knockdown of PTEN induces 2-3 folds increase in the population of CDR-producing cells. It is noteworthy that the effects of wtPTEN and shPTEN on CDR formation resembles closely to that of wtp53 and shp53, respectively (Fig. 2B), strongly suggesting that PTEN is a collaborator or positive mediator of p53. This possibility was further investigated by determining if the effects of p53 and PTEN on CDR formation are positively connected. As shown in Fig. 5B, activation of p53 by doxorubicin nullifies the heightened CDR formation induced by PTEN knockdown; and in parallel, overexpression of wtPTEN reduces the CDR formation capacity of shp53 cells down to that of control cells (Fig. 5B). These data strongly suggest that PTEN acts downstream of p53 in the inhibition of CDR formation in RASM cells.

Next, we asked whether PTEN inhibits CDR formation by down regulating Rac and Cdc42. To this end, we transiently transfected the shPTEN-expressing immortalized RASM cells with Rac1 or Cdc42 siRNAs. As shown in Fig. 5C, knock-down of Rac1 and Cdc42 nullify the enhanced potential of CDR-formation induced by shPTEN. Together these data show that p53 inhibits CDR formation by upregulating PTEN, which in turn down regulates Rac1 and Cdc42.

### The lipid phosphatase activity of PTEN plays a dominant role in the inhibition of CDR generation

PTEN is better known as a lipid phosphatase, however, it also possesses protein phosphatase activity that appears to play a role in the regulation of cell invasion [32], [33]. To determine which activity of PTEN is responsible for the regulation of CDR, three different mutants of PTEN were used. The C124SPTEN construct is both lipid and protein phosphatase-dead, G129EPTEN has lost its lipid phosphatase activity but retains protein phosphatase activity, and Y138LPTEN has only lipid phosphatase activity [34], [35]. Stable retroviral RASM cell lines were created expressing these mutants. In comparison to the PTEN knockdowns, the lipid/protein phosphatase dead mutant (C124SPTEN) and lipid phosphatase-dead mutant (G129EPTEN) have lost most of their ability to suppress the formation of CDR (Fig. 5D). This suggests that the lipid phosphatase activity plays a major role in the inhibition of PDGF-induced CDR formation and is consistent with the observation that overexpression of the lipid phosphatase mutant (Y138LPTEN) restores CDR and lamellipodia formation potentials similar to that of control cells, but has a less suppressive effect as overexpressing wtPTEN. These data indicate that both lipid and protein phosphatase activities are required for maximal suppression of PDGF-induced lamellipodia and CDR formation, but the lipid phosphatase activity clearly plays a dominant role.

## Discussion

In this study, we have shown that the tumor suppressor p53 plays a significant role in suppressing PDGF-induced CDR formation in RASM cells. These results further support the emerging theme that p53, besides its well-known roles in cell cycle regulation, senescence and apoptosis, is a crucial regulator of actin cytoskeleton remodeling and a suppressor of cell migration and invasion.

Analyses of immortalized RASM cells expressing wtp53 and p53-targeted shRNAs allow us to identify Rac and Cdc42 as key mediators of p53 inhibition of CDR formation. This finding is consistent with reports that p53 down-regulates Rac and Cdc42 in cell migration, although the mechanisms involved were not defined [13], [21], [22]. Rac regulates cell migration and CDR formation by binding to and activating Scar/WAVE, members of the WASP family leading to the activation of the Arp2/3 complex and actin polymerization and it has been reported that loss of WAVE1, not WAVE2, inhibits CDR formation [14], [36]. However, the Cdc42 effector, N-WASP, but not WAVE1/2(Scar1/2), was shown to be important in CDR generation in mouse embryonic fibroblasts [15]. Here, we have shown that N-WASP is the major, positive mediator of PDGF-induced CDR formation in RASM cells; in comparison, WAVE1/2 likely play a minor role, at least in RASM cells. These results raise an interesting question: what is the downstream effector(s) of Rac in CDR formation, if WAVE1/2 does not play a significant role? A possible candidate is one or more members of the p21-activated kinase (PAK) family that has been shown to be involved in cell migration, podosome formation and cytoskeleton remodeling [29], [37], [38].

We have made many attempts to directly correlate p53 expression, PDGF-induced CDR formation and Rac1/Cdc42 activation using the CRIB domain of PAK in pull-down assays. In our hands, we consistently observed a small, but not statistically significant, increase in global Rac activities in PDGF-treated cells. This is not too surprising if activation of Rac/Cdc42 by PDGF leading to CDR formation is confined locally to the CDR, global increase in Rac/Cdc42 activities may be too small to be analysed, considering the inherent experimental errors of pull-down assays in general. To circumvent this, we have used imaging data in Fig. 3F to show that activated Rac is indeed localized to CDRs. We have also shown that p53 up-regulates PTEN, which is well-documented to antagonize PI(3,4,5)P_3_ production, and activation of GEFs for Rac and Cdc42, thus providing a possible mechanism by which p53 down-regulates Rac and Cdc42. However, we have not determined the identity of specific GEFs downstream of the p53-PTEN axis, but it would be of interest to investigate whether DOCK1, the atypical GEF of Rac1 shown to be required for CDR formation, is a p53 target [39]. The lipid phosphatase activity of the tumor suppressor, PTEN, negatively regulates the PI3K-Akt-PDK1 pathway by specifically hydrolyzing the 3′ site of phosphatidylinositol-phosphates, PI(3,4,5)P_3_ and PI(3,4)P_2_. Recent studies suggest that PTEN also possesses protein phosphatase activity that has been implicated in the regulation of cell migration [34] . Here we have shown that both lipid and protein phosphatase activities of PTEN are required for maximal effect in p53-mediated CDR inhibition; however, the lipid phosphatase activity clearly plays a dominant role. This finding is different from that observed for p53-PTEN in podosome inhibition, where both lipid and protein activities of PTEN are equally important [32]. While the role of lipid phosphatase activity of PTEN in antagonizing the PI3K/Akt axis is well established, the mechanisms of its protein phosphatase in cell migration is not clear due to a lack of known substrates *in vivo*.

As shown here and in other reports [5], [6], [40], most cells only produce a single round of CDR even when subjected to prolonged PDGF stimulation. Desensitization of cells to prolonged growth factor stress has been shown to be a result of internalization of the receptors including EGFR and PDGFR by clathrin-coated pits, leading to degradation via the C-Cbl-mediated ubiquitination pathway [41]–[43]. It is conceivable that internalization of PDGFR may also be achieved by CDR, which has been shown to internalize surface receptors such as integrins and growth factor receptors by macropinocytosis [7], offering a rapid and global mechanism to blunt PDGF stress. In addition, PDGFR has been shown to be a transcription target of mutant p53 and may also contribute to its down-regulation [44], [45]. This is complicated by the finding that PDGF also suppresses p53 expression, thus creating a negative feed-back mechanism. These are attractive possibilities that may account for the interplay among p53, PDGFR and CDR not only in cell migration, but also in stress response signaling.

Our previous work has shown that Src-induced formation of podosome rosettes in RASM cells and invasion of ECM are also down regulated by p53 involving PTEN, Akt and caldesmon [18], [19], [23], [26]. It is likely that p53 may act on multiple effectors of cytoskeleton remodeling in a concerted manner to down regulate cell migration and invasion. For instance, it was recently reported that lengthy treatment of vascular smooth muscle cells with PDGF for 24 hours inhibits p53 and miR-143/145 resulting in podosome formation [23], [46]. It would be of interest to investigate whether regulation of CDR formation by p53 also involves miR-143/145, which regulates the switch of vascular smooth muscle cells from the contractile to synthetic phenotypes [47]. Integrin, which plays a pivotal role in cell migration and invasion by virtue of its inside-out and outside-in signaling [48]–[50], is another possible target of p53 in the regulation of CDR formation. Although integrin has not been shown to be a transcriptional target of p53, there is good evidence that p53 down regulates integrin signaling in cell migration [20], [51].

## Supporting Information

Movie S1 **RASM cells produce PDGF-induced circular dorsal ruffles.** Time-lapse movie of RASM cells treated with 20ng/mL PDGF display circular dorsal ruffles.
(ZIP)
